# Accurate prediction of functional, structural, and stability changes in *PITX2* mutations using *in silico* bioinformatics algorithms

**DOI:** 10.1371/journal.pone.0195971

**Published:** 2018-04-17

**Authors:** Morteza Seifi, Michael A. Walter

**Affiliations:** Department of Medical Genetics, Faculty of Medicine & Dentistry, University of Alberta, Edmonton, Alberta, Canada; NIDCR/NIH, UNITED STATES

## Abstract

Mutations in *PITX2* have been implicated in several genetic disorders, particularly Axenfeld-Rieger syndrome. In order to determine the most reliable bioinformatics tools to assess the likely pathogenicity of *PITX2* variants, the results of bioinformatics predictions were compared to the impact of variants on *PITX2* structure and function. The MutPred, Provean, and PMUT bioinformatic tools were found to have the highest performance in predicting the pathogenicity effects of all 18 characterized missense variants in *PITX2*, all with sensitivity and specificity >93%. Applying these three programs to assess the likely pathogenicity of 13 previously uncharacterized *PITX2* missense variants predicted 12/13 variants as deleterious, except A30V which was predicted as benign variant for all programs. Molecular modeling of the *PITX2* homoedomain predicts that of the 31 known *PITX2* variants, L54Q, F58L, V83F, V83L, W86C, W86S, and R91P alter PITX2’s structure. In contrast, the remaining 24 variants are not predicted to change PITX2’s structure. The results of molecular modeling, performed on all the *PITX2* missense mutations located in the homeodomain, were compared with the findings of eight protein stability programs. CUPSAT was found to be the most reliable in predicting the effect of missense mutations on *PITX2* stability. Our results showed that for *PITX2*, and likely other members of this homeodomain transcription factor family, MutPred, Provean, PMUT, molecular modeling, and CUPSAT can reliably be used to predict *PITX2* missense variants pathogenicity.

## Introduction

Paired-like homeodomain transcription factor 2 (*PITX2*, RefSeq NM 000325.5, MIM# 601542) is located at 4q25 and is expressed in the developing eye, brain, pituitary, lungs, heart, and gut [1]. Mutations in human *PITX2* or the forkhead box transcription factor C1 (*FOXC1*; 6p25, RefSeq NM 001453.2, MIM# 601090) underlie the autosomal dominant disorder called Axenfeld-Rieger syndrome (ARS; MIM# 602482) [2–5]. ARS is a full penetrant, but clinically and genetically heterogeneous disorder characterized by developmental anomalies involving both ocular and non-ocular structures [6]. To date, 87 mutations within the *PITX2* gene have been identified including deletions, insertions, splice-site mutations, and coding region frameshift, nonsense and missense mutations [7–13].

Identifying new disease-associated variants is becoming increasingly important for genetic testing and it is leading to a significant change in the scale and sensitivity of molecular genetic analysis [14]. One of the most frequent approaches for detecting novel variants in target genes is using direct gene sequencing. However, due to increasing number of newly identified missense variants, it is often difficult to interpret the pathogenicity of these variants as not all the mutations alter protein function, and the ones that do may also have different functional impacts in disease [15,16]. Thus, prior to detailed analyses, novel variants cannot be easily classified as either deleterious or neutral, because of their unknown functional and phenotypic consequences. Therefore, further research should be conducted to validate the genetic diagnosis when a novel missense variant is discovered. Preferably, *in vitro* characterization of novel variants should be undertaken; however, due to facility limitation, it is often not practicable to experimentally verify the impact of large number of mutations on protein function [17]. Another robust approach to substantiate the pathogenicity is using animal models by generating the homologous mutation that recapitulates the human phenotype; but, similar to *in vitro* studies, these are time-consuming, labor-intensive, difficult and expensive, making this approach unfeasible to experimentally determine the pathogenicity effects of all novel identified variants [18]. To circumvent the above mentioned limitations and to provide fast and efficient methods for predicting the functional effect of nonsynonymous variants on protein stability, structure, and function, several computational tools have been developed [19–21].

Protein stability and structure are key factors affecting function, activity, and regulation of proteins. Conformational changes are necessary for many proteins’ function and disease-causing variants can impair protein folding and stability. Missense variants are also capable of impairing protein structure, likely by affecting protein folding, protein-protein interaction, solubility or stability of protein molecules. The structural effect of mutational changes can be examined *in silico* on the basis of three-dimensional structure, multiple alignments of homologous sequences, and molecular dynamics [22–24]. Therefore, analysing sequence data *in silico* first and detecting a small number of predicted deleterious mutations for further experimental characterization is a key factor in today’s genetic and genomic studies.

In general, bioinformatics prediction methods obtain information on amino acid conservation through alignment with homologous and distantly related sequences. The most common criteria considered in many bioinformatics programs for predicting the functional effect of an amino acid substitution are amino acid sequence conservation across multiple species, physicochemical properties of the amino acids involved, database annotations, and potential protein structural changes [23,25,26]. As mentioned above, resources for *in vitro* and *in vivo* functional analysis of novel variants are constrained in most clinical laboratories. Therefore, identifying and reporting novel variants that are likely to be pathogenic often requires accurate prediction using computational tools.

In a previous study, we examined the effect of *FOXC1* variants on protein structure and function by combining laboratory experiments and *in silico* techniques. Our results showed that integration of different algorithms with *in vitro* functional characterization serves as a reliable means of prioritizing, and then functional analyzing, candidate *FOXC1* variants [27]. Unlike most previous studies that focused on using only PolyPhen and SIFT to predict the pathogenicity of missense mutations, here, we investigated the predictive value of SIFT, PolyPhen and nine other prediction tools by comparing their predictions to *in vitro* functional data for *PITX2* variants. The bioinformatics programs found to be most reliable were then used to predict the likely consequences of 13 functionally-uncharacterized *PITX2* variants. We also performed molecular modeling on all the *PITX2* missense mutations located in the homeodomain and compared the results with the findings of protein stability algorithms to identify the most reliable tools in predicting the effect of missense mutations on *PITX2* stability. To the best of our knowledge, this is the first study that incorporates the results of functional studies in conjunction with bioinformatics approaches for predicting the pathogenicity of mutations in *PITX2* gene.

## Materials and methods

### Source of missense variants

**Fig 1:**
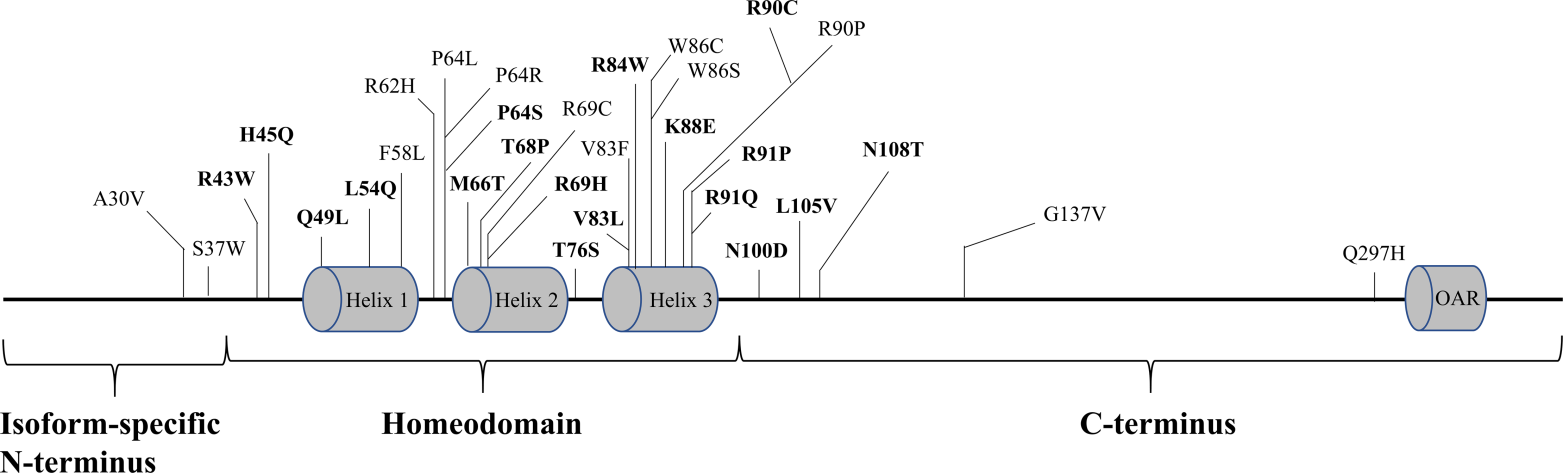
Summary of all 31 known pathogenic missense variants in PITX2. Characterized variants are shown in bold type.

Lists of *PITX2* missense variants were assembled from the previous literature and a search using the ClinVar [28], Human Gene Mutation Database (HGMD) [29], the Genome Aggregation Database (gnomAD), and the single nucleotide polymorphism database (dbSNP). This study found 47 *PITX2* missense variants; 31 of which were described in the literature as being associated with ARS or coronary artery disease (CAD), while the remaining 16 variants, were considered as benign variants (Fig 1). Eighteen of the 31 variants were classified as pathogenic based on functional studies utilizing site-directed mutagenesis, expression studies, and other functional analysis (Table 1). Thirteen of 31 variants were described as associated with ARS and CAD in the absence of functional analyses on *PITX2* structure or function. Sixteen SNPs, with population allele frequencies > 0.0005 were identified from the gnomAD and the ClinVar. Based upon the allele frequency (approximately 10-fold greater than the disease frequency of ARS) these have been considered benign polymorphisms. Nucleotide numbering of the mutations herein indicates cDNA numbering with +1 as the A of the ATG translation initiation codon in the NCBI reference sequence NM_000325.5, while the amino positions are based on the corresponding NCBI reference sequence NP_000316.2. This study is a retrospective case report that does not require ethics committee approval at our institution. All patients’ mutations and phenotypes were obtained from previously published studies.

**Table 1:** Position, effects on protein function and associated phenotype of previously characterised PITX2 missense variants.

No	Variant	Exon	Domain	Phenotype	Effect on protein function	Reference
1	R43W	5	HD	ARS	Reduced DNA-binding and transactivational activity	Idress et al. 2006 [30] Footz et al., 2009 [31]
2	H45Q	5	HD	CHD	Reduced transactivational activity	Yuan et. 2013 [32]
3	Q49L	5	HD	TOF	Reduced transactivational activity	Sun et al. 2016 [33]
4	L54Q	5	HD	ARS	Reduced DNA-binding and transactivational activity	Semina et al. 1996 [34] Amendt et al. 1998 [35]
5	P64S	5	HD	AF	Reduced transactivational activity	Wang et al. 2014 [36]
6	M66T	5	HD	CHD	Reduced transactivational activity	Yuan et al. 2013 [32]
7	T68P	5	HD	ARS	Reduced DNA-binding and transactivational activity	Semina et al. 1996 [34] Amendt et al. 1998 [35] Amendt et al. 2000 [37] Kozlowski and Walter, 2000 [38] Espinoza et al. 2002 [39] Saadi et al. 2001 [40]
8	R69H	5	HD	ARS	Reduced DNA-binding activity	Kulak et al. 1998 [41] Amendt et al. 2000 [37] Strungaru et al. 2007 [42] Kozlowski and Walter, 2000 [38]
9	T76S	5	HD	CHD	Reduced transactivational activity	Wei et al. 2014 [43]
10	V83L	5	HD	ARS	Reduced DNA-binding activity, but increased transactivational activity	Priston et al. 2001 [44]
11	R84W	5	HD	ARS	Reduced DNA binding and transactivational activity	Alward et al. 1998 [45] Amendt et al. 2000 [37] Kozlowski and Walter, 2000 [38] Espinoza et al. 2002 [39]
12	K88E	6	HD	ARS	Reduced DNA binding and transactivational activity	Amendt et al. 2000 [37] Perveen et al. 2000 [46] Saadi et al. 2001 [40]
13	R90C	6	HD	ARS	Reduced DNA binding and transactivational activity	Perveen et al. 2000 [46] Footz et al. 2009 [31]
14	R91P	6	HD	ARS	Reduced DNA binding and transactivational activity	Semina et al. 1996 [34] Amendt et al. 1998 [35] Amendt et al. 2000 [37] Priston et al. 2001 [44] Kozlowski and Walter, 2000 [38]
15	R91Q	6	HD	CHD	Reduced transactivational activity	Wei et al. 2014 [43]
16	N100D	6	Downstream of HD	CHD	Reduced transactivational activity	Wang et al. 2013 [47]
17	L105V	6	Downstream of HD	ARS	Reduced DNA binding activity	Phillips, 2002 [48] Footz et al. 2009 [31]
18	N108T	6	Downstream of HD	ARS	Reduced DNA-binding activity, but increased transactivational activity	Phillips, 2002 [48] Footz et al. 2009 [31]

AF; atrial fibrillation (AF), ARS; Axenfeld-Rieger syndrome (ARS), CHD; congenital heart disease, HD; homeodomain, TOF; tetralogy of Fallot

### Predicting functional impact of missense mutation

*PITX2* amino acid and DNA sequences were obtained from National Center for Biotechnology Information (NCBI) in FASTA format. The functional context of missense mutations was predicted using the default settings of eleven different *in silico* prediction algorithms, SIFT [49], PolyPhen-2 [50], PANTHER-PSEP [51], MutPred [52], MutationTaster [53], Provean [54], PMUT [55], FATHMM [56], nsSNPAnalyzer [57], Align GV-GD [58], and REVEL [59]. These programs were used to analyse 18 functionally characterised *PITX2* missense variants plus 13 additional, functionally uncharacterized *PITX2* missense variants.

SIFT program provides functional predictions for coding variants, based on the degree of conservation of amino acid residues in sequence alignments derived from closely related sequences, collected by PSI-BLAST algorithm [60]. The PolyPhen-2 (Polymorphism phenotyping-2) server predicts possible effect of an amino acid change on the structure and function of a protein using several sources of information such as straightforward physical and comparative considerations [61]. PANTHER-PSEP is a new application that analyses the length of time a given amino acid has been conserved in the lineage leading to the protein of interest. There is a direct association between the conservation time and the likelihood of functional impact [62]. MutPred is a free web-based application that utilizes a random forest algorithm with data based upon the probabilities of loss or gain of properties relating to many protein structures and dynamics, predicted functional properties, and amino acid sequence and evolutionary information [52]. MutationTaster is a tool that combines information derived from various biomedical databases and uses established analysis programs. Unlike SIFT or PolyPhen-2 which work on DNA level, MutationTaster processes substitutions of single amino acids and allows insertions and deletions [53]. Protein variation effect analyzer (PROVEAN) is a web server which uses an alignment-based score approach to generate predictions not only for single amino acid substitutions, but also for multiple amino acid substitutions, and in-frame insertions and deletions [54]. PMUT focuses on the annotation and prediction of pathological variants. PMUT is trained with a massive database of human disease-causing and neutral mutations. PMUT calculates mutational hot spots, which are provided by three different approaches, alanine scanning, genetically accessible mutations, and a very large database of mutation [55]. FATHMM, a web-server software, is able to predict not only the functional consequences coding variants, but also non-coding variants. To assess the large-scale cancer genomic datasets in a short time, FATHMM provides users with unlimited and near instant predictions for all possible amino acid substitutions within the human proteome [56]. sSNPAnalyzer uses the multiple sequence alignment and the 3D structure to evaluate the possible effect of nonsynonymous single nucleotide polymorphism (nsSNP) and also provides extra information about the SNP to aid the interpretation of results, including structural environment and multiple sequence alignment [57]. The Align-GVGD Web-based server uses the biophysical features of amino acids and protein multiple sequence alignments to predict the pathogenicity of missense variants. This tool is an extension of the original Grantham difference to multiple sequence alignments and true simultaneous multiple comparisons [58]. REVEL combines 13 individual prediction tools (MutPred, FATHMM, VEST, PolyPhen, SIFT, PROVEAN, MutationAssessor, MutationTaster, LRT, GERP++, SiPhy, phyloP, and phastCons) as features to predict the pathogenicity of missense variants. REVEL was trained with recently discovered pathogenic and rare neutral missense variants [59]. Please see Table 2 for more information on the prediction tools used in this study.

**Table 2:** Amino acid substitution (AAS) prediction methods used in this study.

Program	Input	Algorithm	Output	URL	Reference
SIFT	PS and AAS, protein sequence alignment and AAS, dbSNP id, or protein id	Uses sequence homology, scores assessment is based on position-specific scoring matrices with Dirichlet priors	Score ranges from 0 to 1, where < = 0.05 is damaging and >0.05 is tolerated	http://sift.jcvi.org/www/SIFT_enst_submit.html	Ng and Henikoff, 2001 [63]
PolyPhen-2	PS and AAS, dbSNP id, HGVbASE id, or protein id	Uses sequence conservation and structure to model location of amino acid substitution, Swiss-Prot and TrEMBL annotation	Score ranges from 0 to 1, where < = 0.05 is benign, and >0.05 is damaging	http://genetics.bwh.harvard.edu/pph2/	Ramensky et al. 2002 [50]
PANTHER-PSEP	PS and AAS	Uses sequence homology; scores are based on PANTHER Hidden Markov Model families	Probably damaging: time > 450my possibly damaging: 450my > time > 200my probably benign: time < 200my)	http://www.pantherdb.org/tools/csnpScoreForm.jsp	Tang and Thomas, 2016 [64]
MutPred	Protein id, PS, or multiple sequence alignment	Prediction is based on one of two neural networks which uses internal databases, secondary structure prediction, and sequence conservation	Score ranges from 0 to 1, where 0 is polymorphism and high scores are predicted to be deleterious/disease-associated	http://mutpred.mutdb.org/	Li et al. 2009 [65]
MutatioTaster	DNA sequence	Predictions are calculated by a naive Bayes classifier, which predicts the disease potential	Prediction is based one of four possible types: a) disease causing: probably deleterious b) disease causing automatic: known to be deleterious c) polymorphism: probably harmless d) polymorphism automatic: known to be harmless	http://www.mutationtaster.org/	Schwarz et al. 2014 [53]
Provean	PS and AAS	Uses an alignment-based score approach to generate predictions not only for single amino acid substitutions, but also for multiple amino acid substitutions, and in-frame insertions and deletions	the default score threshold is currently set at -2.5, in which >-2.5 is neutral, and <-2.5 is deleterious	http://provean.jcvi.org/index.php	Choi and Chan, 2015 [54]
PMUT	PS and AAS, dbSNP, Uniprot or PDB ID of protein	Based on the application of neural networks which uses internal databases, secondary structure prediction, and sequence conservation	Score ranges from 0 to 1, where <0.50 is neutral and >0.50 is disease associated	http://mmb.pcb.ub.es/pmut2017/analyses/new/	Ferrer-Costa et al. 2002 [55]
FATHMM	protein identifier and the amino acid substitution, dbSNP id	Uses sequence homology	The score threshold is set at -2.5, in which >-2.5 is neutral, and <-2.5 is deleterious	http://fathmm.biocompute.org.uk/index.html	Shihab et al. 2013 [56]
nsSNPAnalyzer	Protein sequence in FASTA format and a substitution file denoting the SNP identities to be analyzed	Uses information contained in the multiple sequence alignment and information contained in the three-dimensional protein structure to make predictions.	Normalized probability of the substitution calculated by the SIFT program	http://snpanalyzer.uthsc.edu/	Bao et al. 2005 [57]
Align GV-GD	Protein sequence in FASTA format and a substitution file denoting the SNP identities to be analyzed	Uses biophysical features of amino acids and protein multiple sequence alignments	A value of C > 0 was considered deleterious; otherwise a variant was neutral	http://agvgd.hci.utah.edu/	Tavtigian et al. 2006 [58]
REVEL	Precomputed REVEL scores are provided for all possible human missense variants	Prediction is based on a combination of scores from 13 individual tools	Score ranges from 0 to 1, where <0.50 is neutral and >0.50 is pathogenic	https://sites.google.com/site/revelgenomics/	Ioannidis et al. 2016 [59]

AAS; amino acid sequences, PS; protein sequence, PDB, protein data bank

### Molecular modeling of the mutant protein structure

The NMR structure of the homeodomain of *PITX2* complexed with a TAATCC DNA binding site (PDB: 2LKX) were analyzed by the SWISS-MODEL server (http://www.expasy.org/spdbv/; provided in the public domain by the Swiss Institute of Bioinformatics, Geneva, Switzerland). Model structures of wild-type and mutants were created in Swiss-Pdb Viewer and investigated using the ANOLEA server (http://melolab.org/anolea). For structure predictions of *PITX2*, sequence in FASTA format was obtained from NCBI database (NP_001191327.1).

### Calculating changes in protein stability

Eight different protein stability programs (DUET, SDM, mCSM I-Mutant3.0, MUpro, iPTREE-STAB, CUPSAT, and iStable) were used to predict the effects of missense mutations on the stability of *PITX2* protein. DUET is a web server that uses integrated computational approach to predict effect of missense mutations on protein stability [66]. DUET calculation is based on complementary data regarding the mutation including secondary structure [67] and a pharmacophore vector [68]. SDM, a computational method, has been demonstrated as the most appropriate method to use along with many other programs. SDM assesses the amino acid substitution occurring at specific structural environment that are tolerated within the family of homologous proteins of defined three dimensional structures and change them into substitution probability tables [69]. mCSM relies on graph-based signature concept and predicts not only the effect of single-point mutations on protein stability, but also protein–protein and protein–nucleic acid binding [70]. I-Mutant3.0 is a neural-network-based web server that predicts automatically protein stability changes upon single point protein mutations based on either protein sequence or protein structure. I-Mutant3.0 can predict the severity effect of a mutation on the stability of the folded protein [71]. MUpro is a set of machine learning programs that accurately calculates protein stability alterations based on primary sequence information particularly where the tertiary structure is unrevealed, overcoming a major restriction of previous methods which are based on the tertiary structure [72]. iPTREE-STAB is a web service and mainly provides two function modules of services including discriminating the stability of a protein upon single amino acid substitutions and predicting their numerical stability values [73]. CUPSAT uses protein environment specific mean force potentials (through solvent accessibility and secondary structure specificity) to analyse and predict protein stability changes upon point mutations [74]. iStable, a combined predictor, was designed by using sequence information and prediction data from various element predictors. iStable is available with two different input types: structural and sequential [75]. Please see Table 3 for more information on the stability predictors used in this study.

**Table 3:** Protein stability prediction methods used in this study.

Program	Input	Algorithm	Output	URL	Reference
DUET	Protein structure	Uses SVM regression with a Radial Basis Function kernel, and RSA	Score ranges from negative to positive numbers, where negative number denote destabilizing, and positive number denote stabilizing	http://bleoberis.bioc.cam.ac.uk/duet/	Pires et al. 2014 [66]
SDM	Protein structure	Uses conformationally constrained environment-specific substitution tables (ESSTs)	Score ranges from negative to positive numbers, where negative number denote destabilizing, and positive number denote stabilizing	http://131.111.43.103/prediction	Pandurangan et al. 2017 [69]
mCSM	Protein structure	Uses the concept of graph-based structural signatures	Score ranges from negative to positive numbers, where negative number denote destabilizing, and positive number denote stabilizing	http://biosig.unimelb.edu.au/mcsm/protein_protein	Pires et al. 2014 [70]
I-Mutant3.0	Protein sequence alone or protein structure	Using SVM regression with a Radial Basis Function kernel, and RSA	Score ranges from negative to positive numbers, where negative number denote destabilizing, and positive number denote stabilizing	http://gpcr2.biocomp.unibo.it/cgi/predictors/I-Mutant3.0/I-Mutant3.0.cgi	Capriotti et al. 2006 [71]
MUpro	Protein sequence	Uses feed-forward neural networks and SVMs	A score near 0 means unchanged stability. Score near -1 means high confidence in decreased stability. Score near +1 means high confidence in increased stability	http://www.ics.uci.edu/~baldig/mutation.html	Cheng et al. 2006 [72]
iPTREE-STAB	Protein sequence	Based on the neighboring residues of short window length	Score ranges from negative to positive numbers, where negative number denote destabilizing, and positive number denote stabilizing	http://210.60.98.19/IPTREEr/iptree.htm	Huang et al. 2007 [76]
CUPSAT	Existing PDB structures or custom protein structures	Uses structural environment specific atom potentials and torsion angle potentials	Score ranges from negative to positive numbers, where negative number denote destabilizing, and positive number denote stabilizing	http://cupsat.tu-bs.de/	Parthiban et al. 2006 [74]
iStable	Protein sequence or PDB structure (PDB ID)	Uses SVM	Score ranges from negative to positive numbers, where negative number denote destabilizing, and positive number denote stabilizing	http://predictor.nchu.edu.tw/istable/indexSeq.php	Chen et al. 2013 [75]

RSA; residue relative solvent accessibility, SVM; support vector machine

### Variants classification

Previous analyses of missense variations in different human diseases predicted that the stability margin without any immediate effect on protein fitness is 1–3 kcal mol^-1^ [77–79]. Mutations that reduce the protein stability by >2 kcal mol^-1^ contribute to severe disease phenotypes [80,81]. Therefore, in this study, all variations were classified as predicted to be neutral (-1.5 < ΔΔG < 1.5), stabilizing (ΔΔG > 1.5) or destabilizing (ΔΔG < -1.5).

## Results

### Bioinformatics functional predictions

The protein sequence and/or protein structure with mutational position and amino acid residue of 18 previously functionally characterized pathogenic *PITX2* missense variants, plus 16 SNPs with a population frequency of higher than 0.05% (thus considered benign polymorphisms), were used to test the predictive value of eleven common bioinformatics prediction programs; SIFT, PolyPhen-2, PANTHER-PSEP, MutPred, MutationTaster, Provean, PMUT, FATHMM, nsSNPAnalyzer, Align GV-GD, and REVEL (Table 4 and Table 5). To evaluate the performances of the programs, seven measures (sensitivity, specificity, accuracy, precision, positive predictive value (PPV), negative predictive value (NPV), and Matthews correlation coefficient (MCC)) were calculated by comparing the results of all programs with previously generated functional data.

**Table 4:** Functional characterization vs. bioinformatics programs. Comparison of *in silico* program predictions of degrees of tolerance for 18 functionally characterized *PITX2* missense mutation.

No	Missense variants	SIFT Score	PolyPhen-2 Score	MutPred Score	MutationTaster Score	Provean Score	PANTHER-PSEP Score	PMUT Score	FATHMM Score	nsSNPAnalyzer Score	Align GV-GD Score	REVEL Score
1	R43W	0 (√)	0.003 (×)	0.952 (√)	101 (√)	-7.125 (√)	PD (√)	0.91 (√)	-5.53 (√)	0.00 (√)	C65 (√)	0.599 (√)
2	H45Q	0.21 (×)	1.000 (√)	0.672 (√)	24 (√)	-7.176 (√)	PD (√)	0.87 (√)	-3.86 (√)	0.15 (×)	C15 (√)	0.903 (√)
3	Q49L	0.25 (×)	0.995 (√)	0.598 (√)	113 (√)	-6.498 (√)	PD (√)	0.76 (√)	-3.96 (√)	0.84 (×)	C65 (√)	0.555 (√)
4	L54Q	0 (√)	0.997 (√)	0.959 (√)	113 (√)	-5.598 (√)	PD (√)	0.91 (√)	-6.23 (√)	0.00 (√)	C65 (√)	0.856 (√)
5	P64S	0 (√)	0.999 (√)	0.867 (√)	74 (√)	-7.547 (√)	PD (√)	0.85 (√)	-5.05 (√)	0.00 (√)	C65 (√)	0.908 (√)
6	M66T	0 (√)	0.995 (√)	0.566 (√)	81 (√)	-5.555 (√)	PD (√)	0.88 (√)	-3.64 (√)	0.01 (√)	C65 (√)	0.595 (√)
7	T68P	0 (√)	0.946 (√)	0.854 (√)	38 (√)	-5.094 (√)	PD (√)	0.87 (√)	-3.81 (√)	0.01 (√)	C35 (√)	0.747 (√)
8	R69H	0 (√)	0.007 (×)	0.985 (√)	29 (√)	-4.733 (√)	PD (√)	0.90 (√)	-4.42 (√)	0.00 (√)	C25 (√)	0.752 (√)
9	T76S	0 (√)	0.995 (√)	0.655 (√)	58 (√)	-3.652 (√)	PD (√)	0.89 (√)	-4.01 (√)	0.01 (√)	C55 (√)	0.706 (√)
10	V83L	0.01 (√)	0.902 (√)	0.944 (√)	32 (√)	-2.758 (√)	PD (√)	0.89 (√)	-4.91 (√)	0.14 (×)	C25 (√)	0.812 (√)
11	R84W	0 (√)	0.994 (√)	0.841 (√)	101 (√)	-7.350 (√)	PD (√)	0.88 (√)	-4.01 (√)	0.00 (√)	C65 (√)	0.907 (√)
12	K88E	0 (√)	0.008 (×)	0.828 (√)	56 (√)	-3.800 (√)	PD (√)	0.88 (√)	-3.92 (√)	0.04 (√)	C55 (√)	0.725 (√)
13	R90C	0 (√)	0.957 (√)	0.975 (√)	180 (√)	-7.599 (√)	PD (√)	0.91 (√)	-4.45 (√)	0.00 (√)	C65 (√)	0.816 (√)
14	R91P	0 (√)	0.998 (√)	0.959 (√)	103 (√)	-6.649 (√)	PD (√)	0.91 (√)	-5.73 (√)	0.00 (√)	C65 (√)	0.727 (√)
15	R91Q	0 (√)	0.997 (√)	0.918 (√)	43 (√)	-3.800 (√)	PD (√)	0.91 (√)	-5.71 (√)	0.00 (√)	C35 (√)	0.726 (√)
16	N100D	0.2 (×)	0.863 (√)	0.365 (×)	23 (√)	-4.013 (√)	PD (√)	0.81 (√)	-3.65 (√)	0.20 (×)	C15 (√)	0.861 (√)
17	L105V	0.06 (×)	0.974 (√)	0.788 (√)	32 (√)	-1.894 (×)	PD (√)	0.80 (√)	-3.16 (√)	0.27 (×)	C25 (√)	0.861 (√)
18	N108T	0.24 (×)	0.990 (√)	0.789 (√)	65 (√)	-3.332 (√)	PD (√)	0.68 (√)	-3.05 (√)	0.35 (×)	C55 (√)	0.443 (×)

PD; probably damaging

√ correspond to functional characterization; ×, do not correspond to functional characterization.

**Table 5:** *In silico* analysis of the effect of 16 PITX2 benign variants.

No	Missense variants	SIFT Score	PolyPhen-2 Score	MutPred Score	MutationTaster Score	Provean Score	PANTHER-PSEP Score	PMUT Score	FATHMM Score	nsSNPAnalyzer Score	Align GV-GD Score	REVEL Score
1	P41S	0.25 (√)	0.002 (√)	0.201 (√)	74 (×)	0.325 (√)	PD (×)	0.16 (√)	-2.80 (×)	0.38 (√)	C65 (×)	0.237 (√)
2	Q75P	0.33 (√)	0.000 (√)	0.371 (√)	76 (×)	-0.065 (√)	PD (×)	0.14 (√)	-2.77 (×)	0.27 (√)	C65 (×)	0.503 (×)
3	V81M	0.11 (√)	0.459 (×)	0.063 (√)	21 (√)	-0.023 (√)	PB (√)	0.08 (√)	-2.74 (×)	0.14 (√)	C15 (×)	0.501 (×)
4	A188T	0.68 (√)	0.027 (√)	0.329 (√)	58 (×)	-1.018 (√)	PD (×)	0.04 (√)	-2.86 (×)	0.57 (√)	C55 (×)	0.151 (√)
5	M207V	0.62 (√)	0.069 (√)	0.386 (√)	21 (×)	-1.461 (√)	PD (×)	0.15 (√)	-2.61 (×)	0.47 (√)	C15 (×)	0.185 (√)
6	R203C	0 (×)	0.968 (×)	0.150 (√)	81 (×)	-0.455 (√)	PB (√)	0.03 (√)	-2.51 (×)	0.12 (√)	C65 (×)	0.146 (√)
7	Q193T	0.38 (√)	0.270 (√)	0.196 (√)	84 (×)	0.630 (√)	PD (×)	0.02 (√)	-.2.60 (×)	0.00 (×)	C35 (×)	0.152 (√)
8	Y131D	0 (×)	0.812 (×)	0.121 (√)	35 (×)	-0.010 (√)	PD (×)	0.03 (√)	-1.64 (√)	0.01 (×)	C65 (×)	0.251 (√)
9	G166D	0 (×)	0.557 (×)	0.283 (√)	76 (×)	-0.357 (√)	PB (√)	0.31 (√)	-2.58 (×)	0.01 (×)	C65 (×)	0.224 (√)
10	H151Y	0 (×)	0.512 (×)	0.045 (√)	75 (×)	-2.400 (√)	PD (×)	0.03 (√)	-2.62 (×)	0.01 (×)	C65 (×)	0.169 (√)
11	I138F	0 (×)	0.671 (×)	0.056 (√)	57 (×)	-0.390 (√)	PB (√)	0.2 (√)	-2.69 (×)	0.04 (×)	C0 (√)	0.145 (√)
12	G205S	0.68 (√)	0.017 (√)	0.193 (√)	87 (×)	-0.372 (√)	PB (√)	0.15 (√)	-2.62 (×)	0.78 (√)	C55 (×)	0.147 (√)
13	G186R	0 (×)	1.000 (×)	0.212 (√)	48 (×)	-1.092 (√)	PD (×)	0.08 (√)	-2.86 (×)	0.00 (×)	C65 (×)	0.103 (√)
14	H57Q	0 (×)	0.736 (×)	0.056 (√)	63 (×)	-3.010 (×)	PB (√)	0.03 (√)	-2.55 (×)	0.00 (×)	C15 (×)	0.112 (√)
15	A246D	0 (×)	1.000 (×)	0.440 (√)	44 (×)	-0.769 (√)	PD (×)	0.18 (√)	-2.65 (×)	0.01 (×)	C65 (×)	0.181 (√)
16	G148W	0 (×)	0.844 (×)	0.195 (√)	65 (×)	-0.242 (√)	PB (√)	0.31 (√)	-2.74 (×)	0.04 (×)	C65 (×)	0.109 (√)

PB; probably benign, PD; probably damaging

√ correspond to functional characterization; ×, do not correspond to functional characterization.

**Fig 2:**
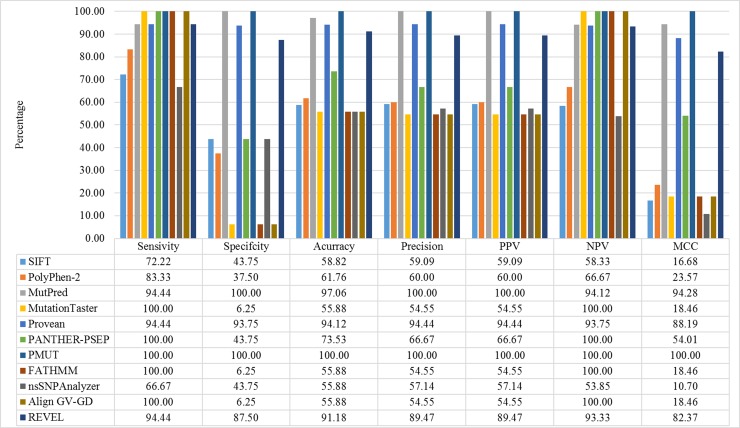
Reliability of eleven *in silico* programs used to analyze all 18 functionally characterized missense variants in *PITX2*. True positives (TP) are missense variants correctly predicted to disrupt PITX2 protein function, and false negatives (FN) are those incorrectly predicted to be benign or tolerated. True negatives (TN) are neutral variants correctly predicted as benign or tolerated and false positives (FP) are neutral variants incorrectly predicted to disrupt PITX2 protein function. The total of variants for all methods was 34, 18 pathogenic variants and 16 benign variants. Values were converted to percentage. Values were converted to percentage. The statistics used were calculated as follows: Sensitivity = *TP/(TP + FN)*; Specificity = *TN/(TN + FP)*; Accuracy = *(TP + TN)/(TP + TN + FP + FN)*; Precision = *TP/(TP + FP)*; Negative predictive value *(NPV) = TN/(TN + FN)*; Positive predictive value (PPV) = TP/(TP + FP); Matthews correlation coefficient *(MCC) = (TP × TN − FP × FN)/-([TP + FP] × [TP + FN] × [TN + FP] × [TN + FN]R)*.

For *PITX2*, MutPred, Provean, and PMUT were the most reliable of the bioinformatics tools in predicting the pathogenicity effects of all 18 functionally characterized missense variants in *PITX2*, with sensitivity and specificity of > 93% (Fig 2). Then, REVEL tool showed high sensitivity and specificity, 94.44% and 87.50%, respectively. Analysis of the sensitivity and specificity SIFT showed that this program had good sensitivity (72.22%) but low specificity (43.75%). Although PolyPhen-2, MutationTaster, PANTHER-PSEP, FATHMM, and Align GV-GD exhibited over 83% sensitivity, they were unable to identify the benign polymorphisms, showing specificity of 37.50%, 6.25%, 43.75%, 6.25%, and 6.25%, respectively. The predictive value of nsSNPAnalayzer was similar to that of SIFT program, with sensitivity and specificity of 66.67% and 43.75%, respectively.

The most reliable programs found in this study’s analyses (MutPred, Provean, and PMUT) were then used to predict the likely pathogenicity of 13 *PITX2* missense variants for which functional testing has not been performed (Table 6). Interestingly, the A30V variant unanimously was predicted as benign by all three programs. The remaining 12 *PITX2* variants were predicted to be disease-associated mutations by all programs.

**Table 6:** Bioinformatics prediction of the degree of tolerance for 13 functionally uncharacterized *PITX2* missense variants.

No	Missense variants	References	Phenotype	Mutpred Score	Provean Score	PMUT Score
1	A30V	Zaidi et al. 2013 [82]	CHD	B, 0.152	B, -0.948	B, 0.10
2	S37W	Yang et al. 2013 [83]	AF	PD, 0.503	PD, -1.074	PD, 0.81
3	F58L	Vieira et al. 2006 [84] D'haene et al. 2011 [85]	ARS	PD, 0.947	PD, -5.560	PD, 0.90
4	R62H	Amendt et al. 2000 [37] Xia et al. 2004 [86]	ARS	PD, 0.856	PD, -4.686	PD, 0.70
5	P64L	Phillips JC, 2002 [48] Weisschuh et al. 2006 [87] Meyer-Marcotty et al. 2008 [88] Dressler et al. 2010 [89]	ARS	PD, 0.973	PD, -9.421	PD, 0.81
6	P64R	Weisschuh et al. 2006 [87]	ARS	PD, 0.944	PD, -8.496	PD, 0.84
7	R69C	Kimura et al. 2014 [90]	ARS	PD, 0.960	PD, -7.575	PD, 0.91
8	V83F	Reis et al. 2012 [91]	ARS	PD, 0.912	PD, -4.643	PD, 0.91
9	W86S	Dandan et al. 2008 [92]	ARS	PD, 0.868	PD, -13.298	PD, 0.91
10	W86C	Reis et al. 2012 [91]	ARS	PD, 0.950	PD, -12.282	PD, 0.91
11	R90P	Phillips JC, 2002 [48]	ARS	PD, 0.960	PD, -6.649	PD, 0.91
12	G137V	Kniestedt et al. 2006 [93]	ARS	PD, 0.816	B, -1.902	PD, 0.61
13	Q297H	Huang et al. 2015 [94]	ARS	PD, 0.682	PD, -3.966	PD, 0.91

AF; atrial fibrillation (AF), ARS; Axenfeld-Rieger syndrome (ARS), ASMD; Anterior segment mesenchymal dysgenesis, B; benign, CHD; congenital heart disease, PD; probably damaging

### Molecular modeling of *PITX2*

Molecular models for the homeodomain of wild-type and variant-containing *PITX2* proteins were designed using threading algorithms to assess impairment of *PITX2* structure by missense variants.

**Fig 3:**
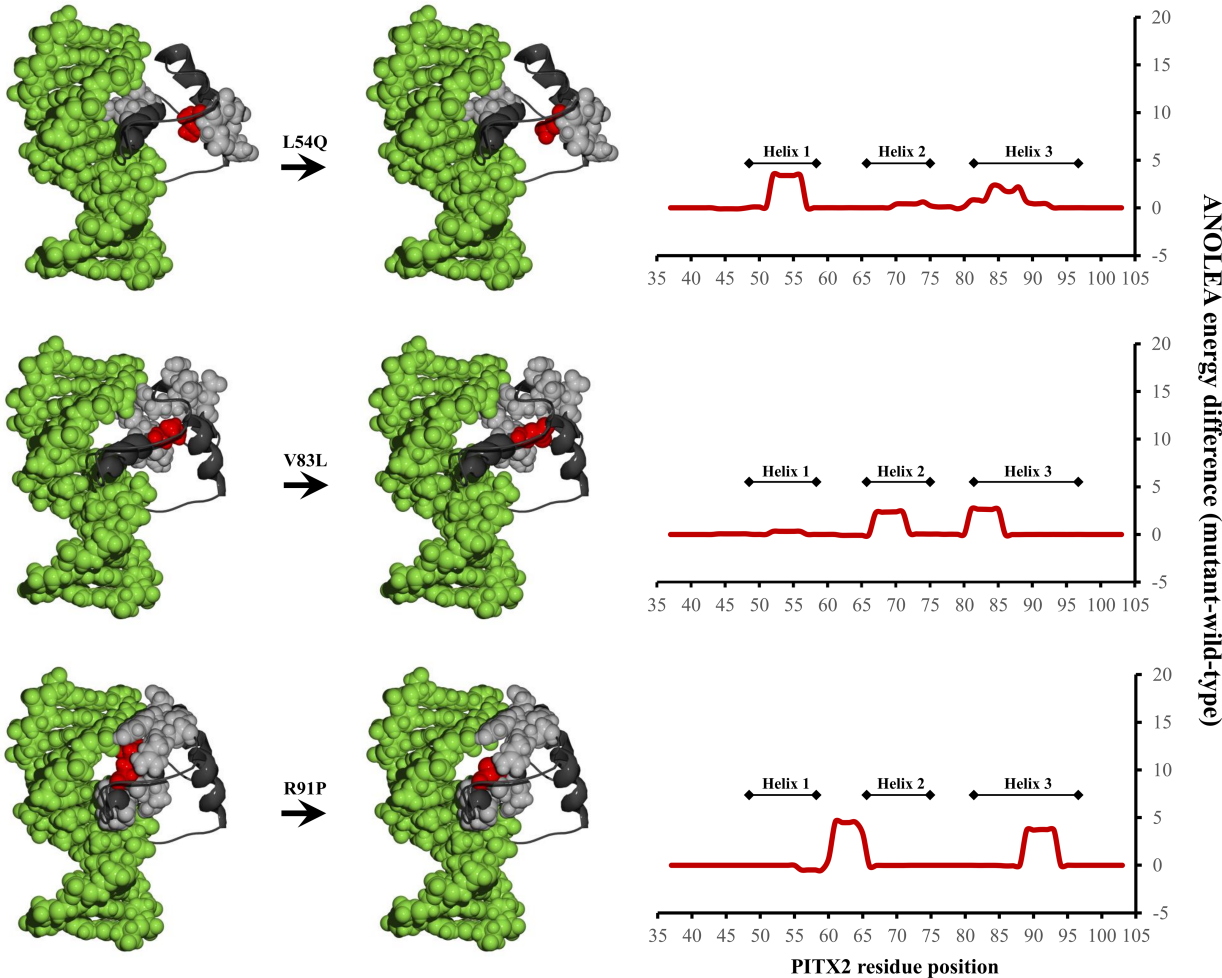
Homology models (left) and scatterplots (right) of *in silico* analyses of the L54Q, V83L, and R91P variants in the *PITX2* gene. The 3D model of *PITX2* is presented with the protein backbone depicted in black ribbon, the co-crystallized DNA binding target in space-filled green model and the mutants positions in red. The wild-type and mutant-equivalent models were analyzed by the atomic nonlocal environment assessment (ANOLEA) server. Peaks on the scatterplots show the positions of amino acids that changed their pseudoenergy state, as a consequence of the mentioned variants.

Three functionally characterised variants, N100D, L105V, and N108T, were excluded from these molecular modeling analyses since they are not located in the homeodomain, which is the only portion of *PITX2* with a known structure. Wild-type amino acids were changed to variant residues to determine putative structural effects of the remaining 15 functionally analysed *PITX2* variants through ANOLEA mean force potential calculations. The molecular modeling identified three mutations as high-risk (L54Q, V83L, and R91P) to change the structure of *PITX2*, particularly in the H1, H2, and H3 subdomains (Fig 3). The R91P variant was predicted to grossly disrupt the non-local amino acid side chain contacts. Similar, although less profound, effects were predicted when L54 and V83 were altered to glutamine and leucine, respectively. In contrast, the remaining twelve amino acid variants showed no predicted substantially altered pairwise interactions, indicating that these missense variants are predicted to have minor or no effects on *PITX2*’s structure (S1 Fig).

**Fig 4:**
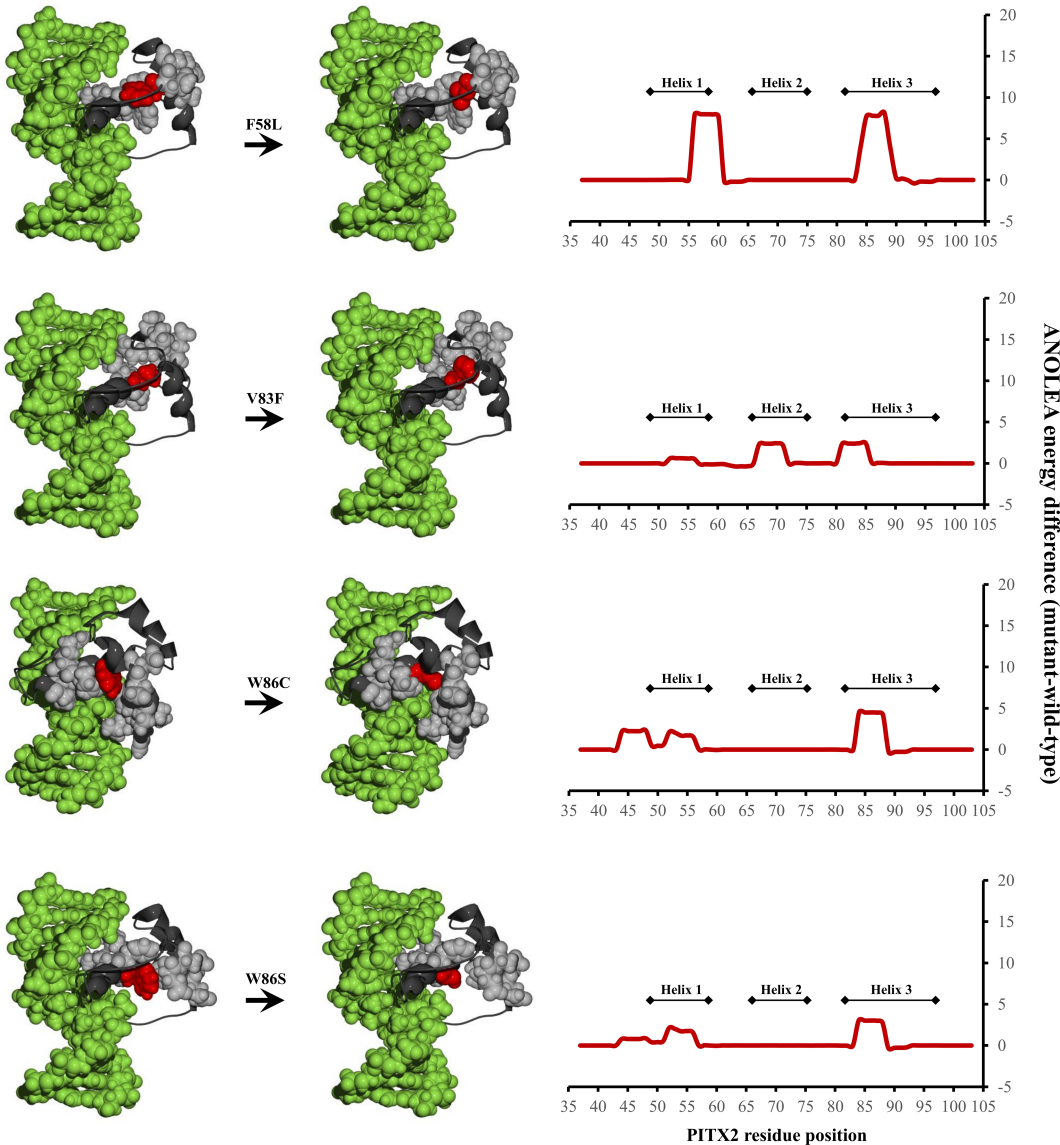
Homology models (left) and scatterplots (right) of in silico analyses of the F58L, V83F, W86C, and W86S variants in the *PITX2* gene. The 3D model of *PITX2* is presented with the protein backbone depicted in black ribbon, the co-crystallized DNA binding target in space-filled green model and the mutants positions in red. The wild-type and mutant-equivalent models were analyzed by the atomic nonlocal environment assessment (ANOLEA) server. Peaks on the scatterplots show the positions of amino acids that changed their pseudoenergy state, as a consequence of the mentioned variants.

Molecular modeling was also performed on the nine functionally uncharacterised *PITX2* missense mutations located in the homeodomain. Four mutations (F58L, V83F, W86C, W86S) were predicted to change the structure of *PITX2* (Fig 4), while, the remaining five variants (R62H, P64L, P64R, R69C, and R90P) were predicted to have minor or no impact on *PITX2*’s structure (S2 Fig).

### Evaluation of the different algorithms in predicting stability changes

To assess the performance of eight different stability predictor programs (DUET, SDM, mCSM, I-Mutant3.0, MUpro, iPTREE-STAB, CUPSAT, and iStable) in predicting the effect of missense mutations on PITX2 protein stability, the change in protein stability (ΔΔG) were computed for all 24 *PITX2* homeodomain variants (15 functionally characterised and 9 functionally uncharacterised mutations) (Table 7).

**Table 7:** Evaluation of stability changes of 15 functionally characterized and 9 functionally uncharacterized PITX2 homeodomain missense variants using eight different protein stability prediction programs.

No.	Variations	DUET	SDM	mCSM	I-Mutant3.0 SEQ	I-Mutant3.0 Structure	MUpro	iPTREE-STAB	CUPSAT	iStable
**Characterised variants**										
1	R43W	-1.773	0.35	-0.97	0.00	-0.13	-0.162	0.0337	-75.87	0.0077
2	H45Q	0.158	-0.15	0.027	0.07	0.17	-0.112	-2.9050	19.1	0.3529
3	Q49L	0.471	0.29	0.186	0.38	0.68	1	0.9422	-5.82	0.6946
4	L54Q*	-2.892	-2.3	-2.73	-1.65	-1.50	-1	-1.8488	3.85	-0.9075
5	P64S	-2.069	-1.27	-1.97	-1.59	-1.57	-1	-1.0233	-45.88	-0.9568
6	M66T	0.444	-0.67	0.181	-1.20	-0.32	-1	1.0943	10.62	-0.1104
7	T68P	-0.359	-0.34	-0.361	-0.90	-0.68	0.155	-1.0594	0.38	0.2945
8	R69H	-2.369	-0.15	-2.147	-1.56	-1.29	-0.633	-1.3667	8.49	-0.7126
9	T76S	-1.35	-0.79	-1.211	-0.69	-0.26	-0.014	0.9377	-16.05	-0.0892
10	V83L*	-0.305	0.09	-0.44	-0.91	-0.72	0.224	-1.3883	-2.72	-0.3060
11	R84W	-1.056	-0.06	-1.125	-0.52	0.41	-0.966	-2.9167	-23.81	-0.1240
12	K88E	-1.759	0.87	-1.777	-0.32	-0.24	-0.024	-0.9691	8.8	-0.1765
13	R90C	-2.014	-0.49	-2.019	-0.86	-0.89	-0.567	-0.6385	-19.6	-0.4268
14	R91P*	-2.225	-2.25	-1.777	-0.82	-0.93	-1	-2.7464	-75.47	-0.6208
15	R91Q	-1.308	-0.08	-1.3	-0.95	-1.04	-1	0.3362	37.96	-0.4777
**Uncharacterised variants**										
1	F58L*	-0.868	0.64	-0.882	-0.69	-0.71	0.446	-1.3492	8.37	0.4267
2	R62H	-1.839	0.2	-1.757	-1.24	-1.17	-0.634	-2.1794	-1.72	-0.6073
3	P64L	-0.55	0.32	-0.845	-0.07	-0.64	-0.260	-4.1000	-5.02	-0.1755
4	P64R	-0.979	-2.07	-0.944	-0.83	-1.09	-0.892	-0.8385	-13.21	-0.5091
5	R69C	-2.183	0.23	-2.107	-1.12	-1.07	-0.183	0.2429	0.51	-0.5278
6	V83F*	-1.437	-1.32	-1.265	-1.16	-1.12	-0.496	-1.3883	-10.94	-0.6159
7	W86S*	-2.327	-2.67	-2.514	-1.64	-1.55	-1	-0.6167	-31.26	-1.0663
8	W86C*	-0.931	-1.57	-1.018	-1.52	-1.40	-0.971	0.6923	-12.15	-0.8733
9	R90P	-1.623	-2.25	-1.319	-0.71	-0.74	-0.346	-2.8825	-23.89	-0.3739

*Predicted by molecular modeling to destabilize the structure and function of PITX2 protein.

Of these eight programs, CUPSAT was the most consistent with the results of our molecular modeling, by identifying 5 of 7 destabilizing mutations that were also predicted to be destabilizing by molecular modeling (V83L, V83F, W86S, W86C, and R91P).

SDM also showed high consistency with the results of our molecular modeling, by detecting 4 of 7 destabilizing mutations that were also predicted to be destabilizing by molecular modeling (L54Q, R91P, W86S, and W86C). DUET, mCSM, and I-Mutant3.0 identified 3 and iPTREE-STAB detected 2 of 7 destabilizing mutations detected by molecular modeling. MUpro and iStable were unable to identify any of the 7 destabilizing mutations predicted by molecular modeling.

## Discussion

Although *in silico* programs are not a substitute for wet-lab experiments, they can provide a supportive role in the experimental validation of disease-associated alleles and can help further diagnostic strategies by prioritizing the most likely pathogenic novel variants.

While many tools are available for assessing the functional significance of variants, determining the reliability of prediction results is challenging. In this context, the current study investigated the combination of experimental findings, molecular modeling, *in silico* mutation prediction programs, and stability prediction software to assess the pathogenicity of *PITX2* missense variants. *In silico* methods that correctly identify deleterious variants do not always inevitably work well for benign predictions. The methods determined by this study to be preferred for analyses of *PITX2* variants were those best able to distinguish both pathogenic and benign variants, thus yielding the highest accuracy.

Our results showed that MutPred, Provean, and PMUT tools were the most accurate in predicting pathogenicity of *PITX2* missense variants (Fig 2). The sensitivity and specificity of these three tools in recognizing *PITX2* disease-causing variants were over 93%, indicating the strong performance of these programs in identifying as pathogenic only *PITX2* variants with significant functional defects. After these three tools, REVEL showed highest sensitivity and specificity, 94.44% and 87.50%, respectively. SIFT showed good sensitivity (72.22%) but low specificity (43.75%). PolyPhen-2, MutationTaster and PANTHER-PSEP, FATHMM, and Align GV-GD demonstrated > 83% sensitivity, but, they were unable to identify the benign polymorphisms, showing the specificity of 37.50%, 6.25%, 43.75%, 6.25%, and 6.25%, respectively. The predictive value of nsSNPAnalayzer was similar to that of SIFT program, with sensitivity and specificity of 66.67% and 43.75%, respectively. Our results showed, therefore, that MutPred, Provean, and PMUT can be utilized with high confidence to test whether or not a *PITX2* missense variant is likely to be deleterious. Interestingly, MutPred was the only *in silico* program that ranked in the top three programs in identifying both pathogenic and benign *PITX2* and *FOXC1* variants [27]. A likely explanation for MutPred’s high ranking is that it evaluates the most factors in making assessments. However, since the number of variants available for testing in this study were small, a larger dataset would confirm that our results are reproducible and generally applicable.

The three programs that were found to be the most reliable (MutPred, Provean, and PMUT) were then used to assess the likely pathogenicity of thirteen *PITX2* missense variants for which functional analyses have not been performed, but which have been associated with ARS or CAD (Table 6). Our results showed that MutPred, Provean, and PMUT predicted as pathogenetic 12/13 of the variants. The A30V variant was scored as non-pathogenetic/benign by all three programs. While it is possible that A30V is an example of a false negative for all three programs, it is likely that this variant is instead benign. Functional testing of the A30V variant is needed to determine which of these possibilities is accurate.

Various intramolecular interactions are involve in stabilizing and folded state of protein, including hydrophobic, electrostatic, and hydrogen-bonding [95–98]. The stability state of a protein is key factor in its proper functionality. In fact, up to 80% of Mendelian disease-causing mutations in protein coding regions are predicted to be caused by altering protein stability [99]. In recent years, due to the availability of high-throughput array-based genotyping methods [100] and next generation sequencing platforms [101,102], a large number of SNPs has been reported. However, the association of missense variants with protein stability has often been difficult to predict. Fortunately, recent advances in computational prediction of protein stability offers potential insight into this question. We used two parallel prediction methods to investigate the possible effects on *PITX2* protein structure and stability of missense variants.

Knowledge of a protein's 3D structure can be used to predict the functionality of protein and the possible impact of variants on protein conformation and structure. We thus first used molecular modelling analyses to assess and compared the total energy difference between native and mutated modeled structure of *PITX2* proteins. The results predicted that while most *PITX2* variants did not dramatically affect the protein tertiary structure, seven variants (L54Q, F58L, V83F, V83L, W86C, W86S, and R91P) altered the total energy level in comparison with the native structure, suggesting that these amino acid substitutions changed the structure of the *PITX2* protein. Molecular modeling of the *PITX2* homeodomain predicted that these variants impair the required energy to maintain the proper folding of helix 1–3 and cause global destabilization of the structure of *PITX2*. These seven amino acids are either invariant (e.g., W86) or highly conserved in the approximately 300 homeobox proteins analyzed, consistent with a pivotal role of these residues in the homeodomain [103–105]. These seven amino acids are tightly packed hydrophobic amino acids responsible for holding helices of the *PITX2* homeodomain together, supporting our molecular modeling predicting that mutations of these amino acids disrupt *PITX2* structure. For F58L, V83F, and V83L, the native wild-type residues and the introduced mutant residues differ in size, probably causing loss of hydrophobic interactions in the core of the protein, particularly involving helix 1–3. For L54Q, W86C, W86S, and R91P, the wild-type residues and the mutant residues are different in both size and charge, likely disturb the local structure of protein thereby altering protein structure and function.

Residues V83, W86, and R91 are located within the third helix which is specifically responsible for binding with the major groove of the DNA [106]. Thus, the prediction that these mutations impair the capacity of this helix to interact with DNA is consistent with this knowledge and with previous functional characterizations that showed reduced DNA-binding capacities of the V83L and R91P mutant *PITX2* proteins [5,107]. Consistent with bioinformatics predictions of deleterious affects of mutation of W86, mutations of the neighboring amino acids (R84W and K88E) have been shown to decrease the ability of the mutant proteins to interact with DNA [39,108].

Residues L54 and F58 are located in helix 1 of the homeodomain, responsible for contacting with the minor groove of the DNA. Molecular modeling of L54Q is consistent with the suggestion that mutations in these highly-conserved residues in helix 1 of the homeodomain might disturb the DNA-protein binding affinity. Our prediction is supported by the fact that changing the leucine to a glutamine (L54Q) disrupts DNA–protein complex, indicating the necessity of leucine at position 54 for *PITX2* binding ability [109]. Thus, consistent with our recent studies on *FOXC1* protein [110], the results of molecular modeling of *PITX2* are strongly consistent with the functional characterization of *PITX2* missense variants.

The results from our molecular modeling analysis were also compared to the predictions of eight stability predictor methods (DUET, SDM, mCSM, I-mutant3.0, MUpro, iPTREE-STAB, CUPSAT, and iStable). Based on our analyses, it appears that CUPSAT performs the best of the seven methods evaluated here in predicting the effect of missense mutations on *PITX2* protein stability, with SDM, DUET, mCSM, and I-Mutant3.0, performing weaker, consistent with the results of previous studies [111,112]. Our results indicate that further studies are required to improve ΔΔG predictions, especially for buried amino acids.

In this study, for the first time, we evaluated the impact of missense variants on *PITX2* stability, structure and function by integrating stability prediction algorithms, bioinformatics mutation prediction tools, and molecular modeling. Our results showed that MutPred, Provean, PMUT, molecular modeling, and CUPSAT are reliable methods to assess *PITX* family missense variants in the absence of laboratory experiments. However, for our analyses, it must be noted that we used sixteen SNPs as non-pathogenetic control variants to investigate the performance of prediction programs. Although we considered SNPs with a population frequency of >0.05% as benign, we cannot formally exclude that these SNPs might have un-documented pathogenic effects on *PITX2*. In addition, while the prediction methods used in this study are not gene-specific, generalization of the performance of these programs to other human genes may be inappropriate without additional study. When assessing the pathogenicity of missense variants, it is necessary to be cautious on depending merely on *in silico* programs without wet-lab experiments. According to standards and guidelines for the interpretation of sequence variants: a joint consensus recommendation of the American College of Medical Genetics and Genomics (ACMG) and the Association for Molecular Pathology, *in silico* predictions only serve as one supporting factor, whereas functional tests are frequently needed to assess the pathogenicity of missense variants. In particular, as per clinical guidelines for the interpretation of single substitution variants, the output of computational tools should be interpreted in the light of functional studies results, population frequency data and segregation in affected families.

## Supporting information

S1 Fig Homology models (left) and scatterplots (right) of *in silico* analyses of functionally characterised variants in the *PITX2* gene. The 3D model of *PITX2* is presented with the protein backbone depicted in black ribbon, the co-crystallized DNA binding target in space-filled green model and the mutants positions in red. The wild-type and mutant-equivalent models were analyzed by the atomic nonlocal environment assessment (ANOLEA) server. Peaks on the scatterplots show the positions of amino acids that changed their pseudoenergy state, as a consequence of the mentioned variants.
(TIF)

S2 Fig Homology models (left) and scatterplots (right) of in silico analyses of functionally uncharacterised variants in the *PITX2* gene. The 3D model of *PITX2* is presented with the protein backbone depicted in black ribbon, the co-crystallized DNA binding target in space-filled green model and the mutants positions in red. The wild-type and mutant-equivalent models were analyzed by the atomic nonlocal environment assessment (ANOLEA) server. Peaks on the scatterplots show the positions of amino acids that changed their pseudoenergy state, as consequence of the mentioned variants.
(TIF)
