# Supplementary figures and images for: Accurate prediction of functional, structural, and stability changes in PITX2 mutations using in silico bioinformatics algorithms
Source: PLoS One. 2018 Apr 17;13(4):e0195971. doi: 10.1371/journal.pone.0195971 (PMC5903617; doi:10.1371/journal.pone.0195971)

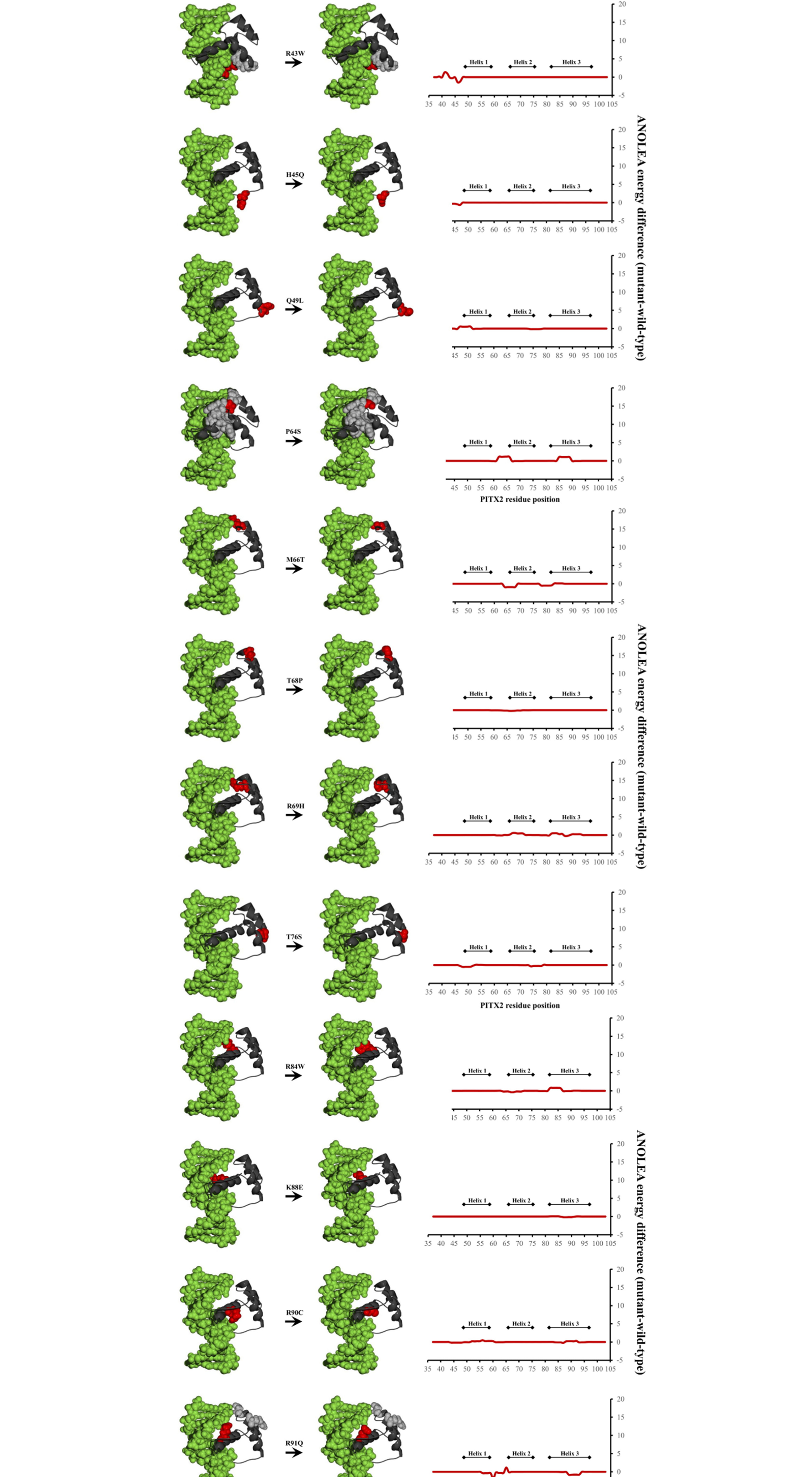

Supplement: S1 Fig — The 3D model of PITX2 is presented with the protein backbone depicted in black ribbon, the co-crystallized DNA binding target in space-filled green model and the mutants positions in red. The wild-type and mutant-equivalent models were analyzed by the atomic nonlocal environment assessment (ANOLEA) server. Peaks on the scatterplots show the positions of amino acids that changed their pseudoenergy state, as a consequence of the mentioned variants. (TIF) [file pone.0195971.s001.tif]

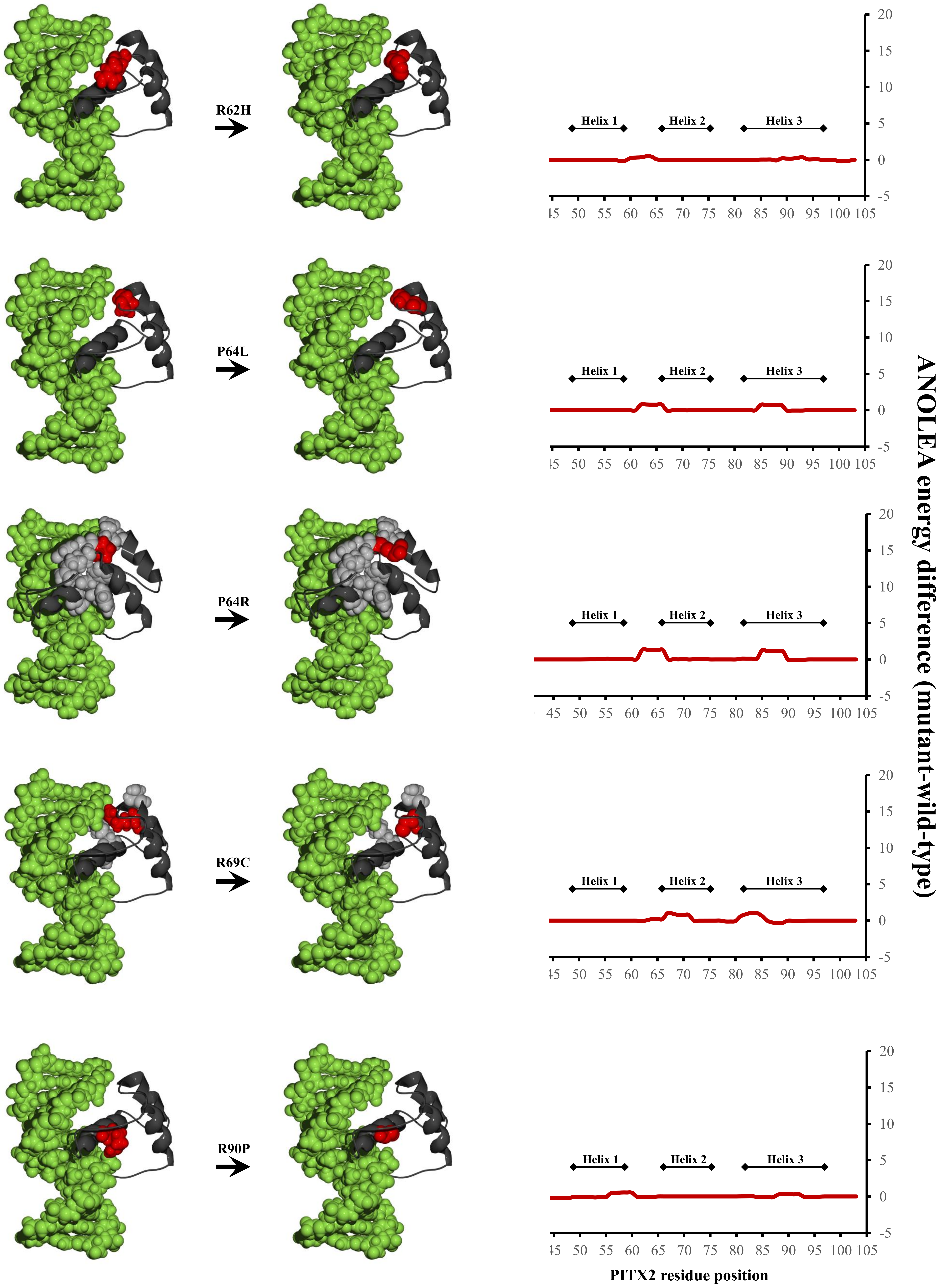

Supplement: S2 Fig — The 3D model of PITX2 is presented with the protein backbone depicted in black ribbon, the co-crystallized DNA binding target in space-filled green model and the mutants positions in red. The wild-type and mutant-equivalent models were analyzed by the atomic nonlocal environment assessment (ANOLEA) server. Peaks on the scatterplots show the positions of amino acids that changed their pseudoenergy state, as consequence of the mentioned variants. (TIF) [file pone.0195971.s002.tif]
